# Preadolescents’ Emotional and Prosocial Responses to Negative TV News: Investigating the Beneficial Effects of Constructive Reporting and Peer Discussion

**DOI:** 10.1007/s10964-017-0675-7

**Published:** 2017-04-19

**Authors:** Mariska Kleemans, Luise F. Schlindwein, Roos Dohmen

**Affiliations:** Behavioural Science Institute, Radboud University, Nijmegen, The Netherlands

**Keywords:** News, Positive (media) psychology, Constructive journalism, Peer discussion, Emotions, Prosocial intentions

## Abstract

Watching news is important for preadolescents, but it may also harm their well-being. This study examined whether applying insights from positive psychology to news production can reduce this potential harm, by reducing negative emotional responses and enhancing positive emotional responses to negative news, and by encouraging prosocial intentions. Moreover, we explored whether peer discussion strengthened these effects. Preadolescents (*n* = 336; 9–13 years old; 48.5% female) were exposed to either constructive (solution-based news including positive emotions) or nonconstructive news. Subsequently, half of the children assigned to the constructive and the nonconstructive condition participated in a peer discussion. The findings showed that exposure to constructive news resulted in more positive emotional responses and less negative emotional responses as compared to nonconstructive news. Moreover, discussing the news with peers led to more positive and less negative emotional responses among preadolescents who watched the nonconstructive newscast, and to more prosocial intentions among preadolescents who watched constructive news. In all, constructive news reporting and peer discussion could function as tools to make negative news less harmful for preadolescents.

## Introduction

Informing preadolescents about what is going on in the world contributes to their socialization into critical and autonomous citizens (van Deth et al. 2011), and can encourage their prosocial intentions and behaviors (de Leeuw et al. 2015). Journalists, politicians, and academics therefore argue that preadolescents’ engagement with news is important (Koltay 2011; Tuominen and Kotilainen 2012). In addition, preadolescents themselves say that they want to follow the news (Alon-Tirosh and Lemish 2014; Carter et al. 2009). However, it has been suggested that exposure to the predominantly negative stories in the news results in increased negative emotional responses, which in turn may lead to anxiety-related behaviors, such as having nightmares (e.g., Buijzen et al. 2007; Riddle et al. 2012). Moreover, prior research indicated that negative, violent television content heightens antisocial behavior in preadolescents (c.f., Browne and Hamilton-Giachritsis 2005). Therefore, the current study aims to examine the usefulness of other framings of the news to inform preadolescents between 9–13 years old about negative news events. To this end, we investigate whether applying insights from positive psychology to news tailored at preadolescents can improve their emotional responses and may encourage prosocial intentions. We use stimuli from a Dutch children’s television news program that targets an audience between approximately 9 and 13 years old (c.f., Walma van der Molen and de Vries 2003), because such tailored newscasts serve as major news source for preadolescents in several Western countries across the world (c.f., Alon-Tirosh and Lemish 2014; Carter et al. 2009; Walma van der Molen and de Vries 2003).

### Positive Psychology in News: Constructive Journalism

Inspired by notions from positive psychology, an innovative kind of news reporting recently emerged in the field of journalism, labeled “constructive journalism” (Gyldensted 2015; Haagerup 2014; McIntyre 2015). Positive psychology focuses on the flourishing of individuals, communities, and societies (Seligman and Csikszentmihalyi 2000), and constructive journalism aims to contribute to this by energizing and inspiring people through news reports (Gyldensted 2015). To reach this aim, journalists are encouraged to give possible solutions for problems in their news stories and to foster a positive perspective on events by including positive emotions (Gyldensted 2015; McIntyre 2015). Therewith, constructive journalism takes distance from the negativity bias that dominates today’s news reporting. Notably, constructive journalism is not about bringing more positive news, but about framing negative stories in a more positive, constructive way (Gyldensted 2015; Haagerup 2014; McIntyre 2015). Given the challenges that producers of newscasts tailored at preadolescents face when reporting about negative events (c.f., Alon-Tirosh and Lemish 2014; Walma van der Molen and de Vries 2003), it is worth exploring whether introducing insights from positive psychology to news (via constructive news reporting) helps to improve preadolescents’ responses to news and to increase their prosocial intentions.

The two principal strategies for creating constructive stories (i.e., solution-based news reporting and including positive emotions in negative stories) are derived from the field of positive psychology. First, Pals’ (2006) idea of coherent positive resolution served as inspiration. Positive coherent resolution involves “the construction of a coherent and complete story of a difficult event that ends positively, conveying a sense of emotional resolution or closure” (Pals 2006, p. 1082). Although not all stories can have a happy end, giving people a sense of narrative completion may help to diminish the negative emotional impact of the story. This may enhance their individual well-being which, in turn, positively affects societal well-being (McIntyre 2015). Applying this to news, a solution-based—instead of problem-based—way of news reporting can provide narrative completion. By presenting solutions and therewith giving the audience the impression that there is hope for the future, news consumers will feel better (Gyldensted 2015; McIntyre 2015). In addition, the presented solutions may serve as mobilizing information (c.f., McIntyre 2015). By showing what potential solutions are, the audience can become inspired to contribute to these solutions.

Second, the broaden-and-build theory (Fredrickson 1998, 2001) is used to conceptualize constructive news reporting. In the broaden-and-build theory, it is argued that positive emotions are able to broaden an individual’s thought-action repertoire, that is the range of potential actions the body and mind are prepared to take. Positive emotions lead to a more positive, broadened mindset and broadened behaviors (e.g., joy may not only cause feelings of happiness, but also spark the urge to play, c.f., Fredrickson 2004). Therewith, it can inspire innovative thoughts and actions in individuals (Fredrickson 1998, 2001). This, in turn, may also be beneficial for communities and societies, because individuals with a broadened repertoire of social and intellectual resources may also have advanced skills in interpersonal relationships. In contrast, negative emotions have an opposite effect, because they narrow one’s thoughts and actions (Fredrickson 1998, 2001). In the context of news, this suggests that stories should include more positive emotions instead of emphasizing particularly negative emotions (McIntyre 2015). News producers can, for example, accomplish this by focusing in the news report on stories of survivors instead of victims.

In all, both solution-based news reporting and the inclusion of positive emotions are expected to improve emotional responses to news and to inspire people to behave in a more prosocial manner (e.g., by offering help). Prior studies among an adult population provide preliminary support for this (Gyldensted 2011; McIntyre 2015). Although the effects of constructive television news reporting are not yet studied among preadolescents, one might expect that constructive stories may also be able to improve emotional responses and prosocial intentions in preadolescents. A study by de Leeuw and colleagues (2015) provides preliminary support for particularly the latter. They found that prosocial news content, in which preadolescents showed how they offered help (i.e., an example of solution-based news), encouraged preadolescents who watched this news to set up a project for a charity organization and to donate money.

### The Additional Effects of Peer Discussion

When studying preadolescents’ reactions to negative news, it is interesting to take peer discussions after news exposure into account. In the Netherlands, where this study was conducted, a substantial amount of preadolescents watches children’s news programs together with their peers in the classroom (c.f., de Leeuw et al. 2015; NOS 2014). After watching, it is reasonable that in-class discussions about the news take place. Until now, no research has been conducted on the impact of such discussions on preadolescents’ reactions to news. There are, however, reasons to expect positive effects of peer discussion after news exposure. According to Wilkinson (2009), discussions in classrooms are defined as collaborative conversations between teachers and students, or between students only, with the purpose of fostering students’ thinking, learning, problem solving, comprehension, and appreciation of the materials.

The importance and impact of discussions among peers has already been stressed by several pioneers in the field of psychology, such as Jean Piaget, Lev Vygotsky, and Harry Stack Sullivan (c.f., Damon 1984). Their theoretical positions provide a conceptual framework for the impact of discussions among peers in the present study. Peer discussions lead to the prevention of misunderstandings, the search for better solutions, discovery learning and creative thinking, and better social competences, such as friendly and fair behavior in interpersonal relationships (Damon 1984; Slavin 2014). Peer-related activities predict preadolescents’ social and academic competences at school, including more frequent displays of prosocial behavior and less antisocial behavior (Wentzel et al. 2009). In addition, they describe peer discussion itself as a form of prosocial behavior, including, for example, helping behavior, cooperation, and providing emotional support. Therefore, in addition to investigating the influence of constructive news reporting, this study also explores the effects of peer discussion on the responses of preadolescents to negative news.

It can be expected that peer discussion alone has a positive influence on the emotions of preadolescents because it fosters, among other things, a better understanding of the news content and leads to friendly and fair behavior (Damon 1984; Slavin 2014). Moreover, based on prior research (Wentzel et al. 2009), one might expect that peer discussion reinforces preadolescents’ prosociality. Although studies in this regard specifically show that longer peer interactions have an effect, we will explore whether holding only one discussion with peers may also affect prosociality. A single peer discussion may not be strong enough to affect behavior, which is why we focus on prosocial intentions, a close cognitive antecedent of behavior (c.f., Ajzen and Fishbein 2005). Because of the assumptions of direct effects of peer discussion on both emotional responses and prosocial intentions, peer discussion is expected to strengthen the effect of constructive news reporting on both the emotional responses of preadolescents and their prosocial intentions.

## Current Study

Constructive news reporting is hypothesized to improve emotional responses to news and to inspire children to be more prosocial, because the constructive elements (i.e., including solutions and positive emotions) create a sense of emotional resolution (c.f., Pals 2006), and may broaden one’s thought-action repertoire in a more positive, innovative way (c.f., Fredrickson 1998, 2001). Therefore, we hypothesize that watching constructive news will lead to less negative emotions and a smaller decrease in positive emotions in preadolescents than watching nonconstructive news (Hypothesis 1), and that watching constructive news will lead to more prosocial intentions among preadolescents than watching nonconstructive news (Hypothesis 2).

In addition, we expect that peer discussion about the news moderates the effect of constructive news reporting on both the emotional responses of preadolescents and their prosocial intentions, because peer discussion in itself fosters, among other things, friendly and fair behavior (Damon 1984; Slavin 2014) and prosociality (Wentzel et al. 2009). Therefore, we hypothesize that discussing news with peers will enhance the expected positive effect of constructive news reporting on preadolescents’ emotional responses to news (Hypothesis 3), and on preadolescents’ prosocial intentions (Hypothesis 4).

## Method

We conducted a between-subjects experiment in which preadolescents were exposed to either constructive or nonconstructive television news. Both before and after exposure to a television newscast, their emotions were measured. Moreover, after watching the news, preadolescents indicated their prosocial intentions. For half of the preadolescents, the experiment ended here. The other half participated in a peer discussion about the news. After this discussion, we again measured their emotions and prosocial intentions.

### Participants

The sample size for the current study was based on an a priori power analysis which was conducted in G*Power 3.1 (Faul et al. 2007, 2009). Assuming an effect size *F* = 0.14, a significance level of *α* = .05, and four participant groups, a total of at least 274 participants was determined. This would provide a power of 80% in order to detect effects. The actual sample consisted of 336 preadolescents (48.5% female; *M*_age_ = 10.60; *SD*_age_ = 1.17). Participants were between the ages of 9 and 13 years, which is in accordance with the target group of children’s TV news (Walma van der Molen and de Vries 2003).

### Procedure

Preadolescents were recruited from four primary schools across the Netherlands. In addition to obtaining active consent to participate from the head of each school and the preadolescents themselves, a letter with a description of the study as well as the request to give passive consent was distributed among the parents of the preadolescents. In this letter, it was further stressed that all information would be treated anonymously and confidentially. Almost all parents (99%) and preadolescents (98%) gave, respectively, passive and active consent.

The data were collected in classrooms, using paper-and-pencil questionnaires. In the beginning of the experimental session, an entire class was randomly assigned to either the experimental (constructive, *n* = 166) or control (nonconstructive, *n* = 170) condition. In addition, within these conditions, classes were also randomly assigned to the peer discussion condition or the condition in which no peer discussion was included. The amount of children that participated in the discussion was roughly the same in the constructive (*n* = 76) and nonconstructive condition (*n* = 81). In the no peer discussion condition, the number of participants was also comparable between the constructive (*n* = 90) and nonconstructive condition (*n* = 89). The mean age of the preadolescents in the peer discussion condition (*M* = 10.59; *SD* = 1.17) was similar to the mean age of the preadolescents that did not participate in a peer discussion (*M* = 10.60; *SD* = 1.18).

After individually filling out the pre-exposure questionnaire—capturing the preadolescents’ demographic characteristics and their emotional state at that moment—preadolescents watched a short news program that either included constructive or nonconstructive news items. Then, preadolescents were asked to, again individually, fill out the second questionnaire (post-exposure) capturing their emotional responses and prosocial intentions after watching the newscast. For approximately half of the classes (*n* = 179 preadolescents), the experiment ended here. These preadolescents were thanked and debriefed.

The other classes (including *n* = 157 preadolescents) participated in a peer discussion. Within each class, preadolescents were divided in groups of three or four. In order to control the direction of the peer discussions held after watching the news, an educational tool called “the placemat method” was used (Craigen and Green 1999; Förrer et al. 2000). A large piece of paper (a placemat) was placed on a table. Because a maximum of four preadolescents participated in a peer discussion group, this paper was divided into four equal areas (corners of the paper, one for each child) and an area in the middle of the paper. First, preadolescents were asked to individually write down their opinion concerning the statement: “Thinking back to the broadcast you just have seen, what thoughts, feelings, and ideas come into your mind first?”. After 2 min, their task was to discuss with the other two/three members of their discussion group what they just had written down in the corner of the paper they were assigned to. After that, they got another 2 min to select the three most important ideas that were discussed and to write them down in the middle of the paper. This was the end of the peer discussion task, after which the participating preadolescents were asked to fill out a last questionnaire (follow up), again capturing their emotional responses and prosocial intentions with regard to the newscast they saw. Afterwards, these preadolescents were also debriefed and thanked for their participation.

### Materials

Together with news producers from the *NOS* *Jeugdjournaal*, a popular children’s TV news program in the Netherlands, two professionally looking versions of the news program were created. We only used real footage that was broadcast in newscasts of the *NOS Jeugdjournaal*. Due to findings of several studies suggesting that news about natural disasters is one of the top categories that frightens preadolescents when watching news (c.f., Cantor and Nathanson 1996; Riddle et al. 2012), participating preadolescents were exposed to news reports about the 2011 tsunami nearby Sendai in Japan. We selected this particular event, because a lot of video material was available. Moreover, the time difference between the moment that the event happened and the data collection played a role. All materials that were used to construct the newscasts were broadcast on television between 12 and 18 March in 2011. The experiment was conducted in the spring of 2016. Therefore, it was unlikely that the preadolescents in our sample saw the original newscasts about the topic or had living memories of the tsunami in Japan that happened 5 years ago.

The structures of the constructive and the nonconstructive newscast were comparable (see Table 1). After the tune that marked the beginning of the program, both versions of the newscast consisted of an opening presenting general information about the tsunami, followed by three news items of approximately the same duration, and an ending and end tune. The opening and the ending were kept the same in both the constructive and nonconstructive version. Not only the same audio information—presenting the most important information about the 2011 tsunami in Japan—but also the same video information was used here. The opening item (1:03 min) contained, for example, information about the number of people killed, the number of people still missing, and about the consequences of the tsunami. In addition, the participants saw the same pictures of the havoc in Japan. Therewith, the magnitude of the disaster was presented in exactly the same way in both news conditions.

**Table 1:** Content of the constructive vs. nonconstructive version of the newscast about the 2011 Tsunami nearby Sendai, Japan

	Constructive	Duration	Nonconstructive	Duration
Opening	Basic information about the tsunami in Japan			01:03
Item 1	Solution-based: Help is coming, people are saved	00:29	Problem-based: Search for missing persons is difficult	00:21
Item 2	Interview with children expressing positive emotions	00:35	Interview with children expressing negative emotions	00:35
Item 3	Focus on survivors	00:20	Focus on victims	00:19
Ending	General closing of the broadcast			00:07

The three items in the middle of each broadcast were different based on the application of elements from positive psychology. The constructive version included solution-based elements and positive emotions (c.f., Gyldensted 2015; McIntyre 2015). Specifically, the first item contained a report about the search for survivors. It showed that several countries sent military aid and tracker dogs to Japan, fostering hope and providing solution-based information. Item 2 showed parts of an interview with two Japanese children who live in the Netherlands. They expressed their happiness about the fact that their grandparents in Japan were fine. Therewith, positive emotions were included in the newscast. Item 3 contained an interview with a Japanese girl describing finding her dog after having him lost for 1 week. Therewith, the focus was on the survivors (instead of victims) and along with this, positive emotions were expressed. The total duration of the constructive newscast was 2 min and 49 s.

The nonconstructive version also included three news items in the middle of the newscast. The topics of these items were comparable to the stories in the constructive newscast. The first item was also about the search for survivors, but particularly emphasized how difficult the search was. It focused on negative consequences of the disaster rather than providing possible solutions. Item 2 showed parts of the interview with the same two Japanese children as used in the constructive newscast. However, in the nonconstructive newscast they expressed their sorrow and sadness about not being able to visit their grandparents in Japan. Item 3 focused on the victims rather than the survivors. A man, standing in the middle of the havoc, reported that there are a lot of victims and that this is of course very sad news. The nonconstructive newscast had a total duration of 2 min and 37 s.

### Measures^1^

In order to measure the emotional responses of the preadolescents, both before and after exposure to the news program as well as after the peer discussion, preadolescents were asked to indicate how they felt on visual analog scales (VAS) ranging from 0 to 100 (100 mm of length). VAS are shown to be a valid, reliable, and sensitive tool for assessing individual subjective feelings (Davey et al. 2007; Lara-Muñoz et al. 2004; Li et al. 2013). Next to that, the VAS is unaffected by the limited test-taking skills of younger participants. Therefore, VAS is frequently used in medical settings (e.g., to let children indicate pain). It is also used to measure emotional responses to media messages. For example, Branton and colleagues (2014) used VAS to measure both positive and negative emotions before and after video gaming. In the current study, we use a VAS in which an emoticon representing the lack of the emotion was displayed on the left end point, whereas on the right end point an emoticon representing the emotion was displayed. Based on Keltner et al. (2014), the four primary emotions—joy, anger, sadness, and fear—that children express already very early in life, were used. In each questionnaire, these and a synonym for each emotion (respectively happiness, madness, sorrow, and anxiety) were included in order to measure the preadolescents’ emotional responses as extensive as possible.

Then, principal component analyses (PCAs) for the two positive emotions and the six negative emotions measured before the newscast, after the newscast, and after peer discussion were conducted separately. Across all testing moments, the PCAs and reliability analyses presented comparable results. The Kaiser–Meyer–Olkin (KMO) measure was in all cases greater or equal to 0.50. Therefore, it verified sampling adequacy for both the positive emotions and negative emotions. Moreover, Bartlett’s test of sphericity was always significant (*p* < .001). We therefore assumed that the correlations between the items were large enough to conduct the PCAs. Both the criterion of component loadings greater than 0.60, and the criterion of dimensions with eigenvalues greater than 1 proposed one component for each PCA (c.f., Kline 1994). Cronbach’s alpha for each PCA was sufficient, ranging between *α* = .79 and *α* = .93 (see Table 2). Based on this, the following four variables were constructed.

**Table 2:** Descriptive statistics for positive and negative emotional responses per testing moment

	Testing moment^a^	Cronbach’s *α*	Mean	SD
Positive emotions	1	.79	80.90	19.85
	2	.90	60.29	30.07
	3	.87	68.05	29.20
Negative emotions	1	.81	6.95	10.91
	2	.85	15.69	17.00
	3	.93	12.24	18.05

^a^ 1 = pre-exposure (*n* = 336), 2 = post-exposure (*n* = 336), 3 = follow up (*n* = 157)

#### Positive emotions

A variable for positive emotions was constructed by, first, subtracting the mean scores on both the happy and joyful items after and before exposure to the newscast. Second, based on these two pre-post difference scores, a mean score for positive emotions was calculated. This variable thus indicated the change in positive emotions caused by the exposure to the news. The negative mean score for this variable (*M* = −20.38; *SD* = 28.09) indicates that positive emotions decreased after exposure to the newscast.

#### Negative emotions

The variable negative emotions was constructed by calculating pre-post difference scores for each of the six negative emotions (i.e., subtracting the scores on how angry, mad, sad, sorrowful, scared, and anxious preadolescents felt after and before the newscast). Based on these six pre-post difference scores, a mean score for negative emotions was calculated, indicating how negative emotions were influenced by exposure to the newscast. As the mean score (*M* = 8.48; *SD* = 15.16) shows, participants experienced an increase in negative emotional feelings after exposure to the news.

#### Final level of positive emotions

To investigate the influence of peer discussion on preadolescents’ emotional responses, we constructed another variable for positive emotions—in which we combined the scores on the positive emotions of the preadolescents who participated in a peer discussion after having watched the newscast (follow up measure) with the answers of the preadolescents who did not discuss after the newscast (post-exposure measure). We refer to this variable as final level of positive emotions, because they represent the level of positive emotions that preadolescents experienced at the last moment they were measured (*M* = 64.29; *SD* = 29.94).

#### Final level of negative emotions

A similar procedure as described before was used to construct a variable representing the final level of negative emotions. The levels of negative emotions of participants who did not participate in a peer discussion (measured directly after exposure to the newscasts) were combined with the levels of negative emotions of the participants after participating in the peer discussion (follow up measures) to indicate their negative emotional feelings at the end of the experiment (*M* = 13.55; *SD* = 16.69).

#### Prosocial intentions

Preadolescents’ prosocial intentions were measured with four items derived from McIntyre (2015). With these items, she aimed to measure how confident participants felt to contribute to social change (i.e., perceived self-efficacy). We believe that the items are also useful to measure prosocial intentions, because they reflect prosocial activities that people can perform to offer help. We tailored the items to the content of the newscast used in the current study and adapted the response categories from totally unconfident/confident to totally disagree/agree to make the items useful for the current study.

To measure prosocial intentions, preadolescents were asked to indicate on a 4-point scale (ranging from 1 = *totally disagree* to 4 = *totally agree*) whether they: (1) thought nothing could be done to save the people in Japan, (2) wanted to help the people in Japan, (3) wanted to give money to aid organizations in order to help the people in Japan, and (4) wanted to start a campaign to help the people in Japan. These questions were included in the questionnaires both after exposure to the newscast and after peer discussion.

First, a principal component analysis (post-exposure measures) indicated that the first item (after reversing) did not fit the other three questions because the factor loading was extremely low (.108). Moreover, the content of this item does not represent a clear (non)prosocial intention. We therefore excluded this item from the analysis. Again, a PCA was conducted with the remaining three items. The KMO measure verified sampling adequacy (>.500), and Bartlett’s test of sphericity (*p* < .001) confirmed that the correlations between the items were large enough to conduct the PCA. The factor loading of the item “I want to help the people in Japan” was only 0.590, indicating that the criterion of component loadings >.60 was only partly reached. However, the criterion of dimensions with eigenvalues >1 still yielded one component for the PCAs. Moreover, Cronbach’s alpha was acceptable (*α* = .75). Therefore, the variable prosocial intentions was constructed by calculating the mean score on the three remaining items (*M* = 2.46; *SD* = .99).

#### Final prosocial intentions

Additionally, we also constructed a variable for final prosocial intentions in order to investigate the influence of peer discussion. This variable consisted of the prosocial intentions that the preadolescents who did not hold a discussion had after having watched the broadcast (post-exposure measure), combined with the prosocial intentions that the preadolescents who did hold a peer discussion had when measuring follow up scores after peer discussion (*M* = 2.43, *SD* = .95).

### Analysis Procedure

The data in this experiment have a nested nature (children are nested in classes, and these classes are nested in schools). However, because of the relatively low number of classes (*n* = 14) and schools (*n* = 4), multilevel analysis is not preferred to use for the analyses (c.f., Hox 2010). Therefore, analyses of variances (ANCOVA) were conducted to test how the news stories affected responses of preadolescents to news, regardless of class and school. To test hypotheses 1 and 2, a between-subjects design with news condition (constructive vs. nonconstructive) as factor was used. Positive emotions, negative emotions, and prosocial intentions were separately added as dependent variables to this model. To test the moderating influence of peer discussions (H3 and H4), the final level of positive emotions, final level of negative emotions, and final prosocial intentions were used as dependent variables in a 2 (news condition: constructive vs. nonconstructive) × 2 (peer discussion: participation vs. no participation) between-subjects model. In all analyses, age and sex were included as covariates. Each hypothesis was tested at *α* = .05 level (two-tailed); Cohen’s *d* is used to indicate the effect sizes.

## Results

### Effects of Constructive News Reporting on Emotions and Prosocial Intentions

**Fig. 1:**
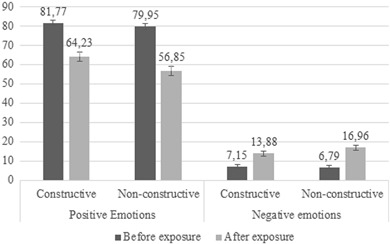
Mean scores of level of positive emotions and negative emotions per news condition before and after exposure

Hypothesis 1 predicted that watching constructive news would lead to more positive and less negative emotional responses among preadolescents than watching nonconstructive news. The analyses revealed that positive emotions decreased after exposure (i.e., negative mean scores for the positive emotions variable), which was expected because the newscast reported about a negative event. However, as the main effect of news condition showed, this decrease was indeed significantly smaller in the constructive condition (*M* = −17.54; *SD* = 26.23) compared to the nonconstructive condition (*M* = −23.10; *SD* = 29.60), *F*(1319) = 3.916, *p* = .049, *d* = .199. Moreover, also in line with our expectation as formulated in the first hypothesis, the main effect of news condition for negative emotions was significant, *F*(1319) = 5.617, *p* = .018, *d* = .229. Watching the constructive newscast led to a significantly lower increase in negative emotions (*M* = 6.72; *SD* = 14.16) than watching the nonconstructive newscast (*M* = 10.17; *SD* = 15.92). This implies that the overall emotional responses of preadolescents to the news were less negative after having watched the constructive newscast compared to the nonconstructive newscast (see Fig. 1). This provides support for the first hypothesis.

Hypothesis 2 predicted that after having watched the constructive newscast, preadolescents would have more prosocial intentions compared to preadolescents who watched the nonconstructive newscast. However, the results did not reveal a significant main effect of news condition on prosocial intentions, *F*(1328) = 1.087, *p* = .298, *d* = .132. Because preadolescents did not differ in their prosocial intentions after watching constructive (*M* = 2.53; *SD* = 1.07) vs. nonconstructive (*M* = 2.40; *SD* = .89) news, the second hypothesis was not supported.

### Effects of Peer Discussion

**Fig. 2:**
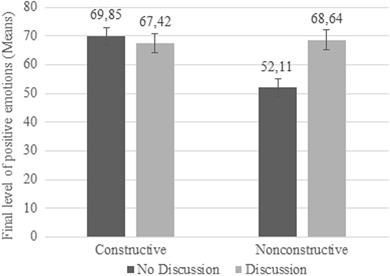
Mean scores of final level of positive emotions for news condition by peer discussion

Hypothesis 3 predicted that discussing news with peers would enhance the positive effects of constructive news reporting on preadolescents’ emotional responses to news. The analysis first revealed that there was a significant main effect of peer discussion on final level of positive emotions, *F*(1329) = 5.074, *p* = .025, *d* = .238. Preadolescents who participated in a discussion had more positive emotions at the end of the study (*M* = 68.05; *SD* = 29.20) than preadolescents who did not participate in a discussion after watching the news (*M* = 60.98; *SD* = 30.27). In addition, there was a significant interaction between peer discussion and news condition on positive emotions, *F*(1329) = 10.746, *p* < .001. Post-hoc *t*-tests showed that preadolescents who watched the nonconstructive broadcast and participated in a discussion displayed more positive emotions than preadolescents who did not participate in a peer discussion after watching the nonconstructive newscast, *t*(168) = −3.602, *p* < .001; *d* = .555 (see Fig. 2). However, preadolescents who watched the constructive newscast and participated in a discussion did not differ in their positive emotions compared to preadolescents who did not participate in a discussion afterwards, *t*(163) = .548, *p* = .584, *d* = .086.

**Fig. 3:**
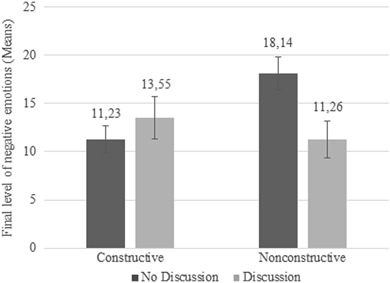
Mean scores of final level of negative emotions for news condition by peer discussion

With regard to the final level of negative emotions, we did not find a main effect of peer discussion, *F*(1327) = 2.063, *p* = .152, *d* = .146. However, we again found a significant interaction between peer discussion and news condition, *F*(1327) = 10.712, *p* = .001. With post-hoc *t*-tests, we investigated the two news conditions separately (see Fig. 3). By looking at the preadolescents in the nonconstructive condition only, we found that preadolescents who participated in a discussion indicated significantly less negative emotions than preadolescents who did not participate in a discussion, *t*(167) = 2.657, *p* = .009, *d* = .411. We did not find a significant difference in negative emotions between preadolescents in the constructive condition who participated in a discussion and preadolescents who did not, *t*(162) = −.809, *p* = .420, *d* = .127. In conclusion, hypothesis 3 is not supported. Peer discussion does not enhance the effects of constructive news on emotions. However, peer discussion showed to be helpful for preadolescents who were exposed to the nonconstructive newscast, because preadolescents who discussed the news with peers have less negative and more positive emotional responses compared to preadolescents who did not participate in a peer discussion after watching nonconstructive news.

**Fig. 4:**
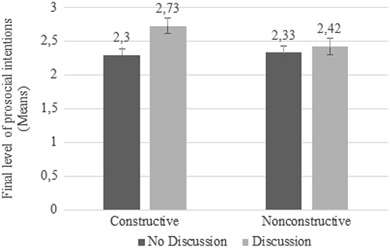
Mean scores of final prosocial intentions for news condition by peer discussion

With regard to the effect of peer discussion on prosocial intentions (H4), a significant main effect was found, *F*(1323) = 4.651, *p* = .032, *d* = .273. Preadolescents who participated in a discussion with peers showed more prosocial intentions (*M* = 2.57; SD = 1.02) than preadolescents who did not participate in a peer discussion (*M* = 2.31; *SD* = .88). Although the interaction between peer discussion and news condition was not significant, *F*(1323) = 1.998, *p* = .158, we still decided to conduct post hoc *t*-tests, because our fourth hypothesis focused on the constructive news condition in particular. These tests showed that peer discussion did not affect the prosocial intentions of preadolescents in the nonconstructive condition, *t*(164) = −.583, *p* = .561, *d* = .091 (see Fig. 4). However, we did find a significant difference in prosocial intentions between preadolescents in the constructive condition who participated in a peer discussion and preadolescents who did not, *t*(161) = −3.104, *p* = .002, *d* = .489. In line with hypothesis 4, the prosocial intentions of preadolescents who held a discussion after watching the constructive newscast were significantly higher (*M* = 2.73; *SD* = .95) than the prosocial intentions of preadolescents who watched the constructive newscast but did not participate in a discussion with their peers (*M* = 2.30; *SD* = .84). In all, as shown in Fig. 4, we can conclude that preadolescents who saw the constructive newscast and discussed this news with peers had the most prosocial intentions afterwards.

## Discussion

It is important to inform preadolescents about what is happening in the world through news, because this contributes to their socialization as citizens in society (van Deth et al. 2011). However, exposure to (negative) news stories may lead to negative emotional responses, anxiety-related behaviors (e.g., Buijzen et al. 2007; Riddle et al. 2012), and may also heighten antisocial behavior in preadolescents (c.f., Browne and Hamilton-Giachritsis 2005). Therefore, the current study aimed to examine how to diminish these potential adverse effects from negative television news on preadolescents. Based on insights from positive psychology—in particular, Pals’ (2006) idea of coherent positive resolution and Fredrickson’s (1998, 2001) broaden-and-build theory—it is argued that including solution-based information and positive emotions in negative news stories may make stories more constructive, and therewith less harmful and more inspiring. We experimentally investigated whether these two elements indeed improved preadolescents’ emotional responses to news and whether their prosocial intentions were encouraged by exposing them to a newscast that reports about a tsunami in either a constructive or nonconstructive way. In addition, we examined the role of peer discussion in this regard, because preadolescents frequently watch the news together with peers in classrooms (c.f., de Leeuw et al. 2015; NOS 2014), and because peer discussion in itself may foster friendly and fair behavior (Damon 1984; Slavin 2014) as well as prosociality (Wentzel et al. 2009).

The findings revealed that preadolescents who watched the constructive newscast reported less negative emotional responses compared to preadolescents who watched the nonconstructive newscast. Moreover, because the newscast was about a negative event, preadolescents in both the constructive and the nonconstructive condition reported a decrease in positive emotions. However, this decrease was smaller for preadolescents watching the constructive version of the news. Additionally, preadolescents who watched the nonconstructive newscast and participated in a discussion with their peers displayed more positive and less negative emotions than preadolescents in the same news condition who did not participate in a peer discussion. This beneficial effect of peer discussion was not found in preadolescents who watched the constructive newscast. For the latter group, peer discussions led to more prosocial intentions. In all, this study indicates that constructive reporting of news and peer discussion can function, at least to a certain extent, as tools to make negative news less harmful for preadolescents. Moreover, discussing constructive content of news may increase their prosocial intentions. These results are generally in line with results from studies on constructive journalism among adults, which demonstrated that constructive elements in news stories can increase positive affect in readers and enhance prosocial intentions, such as donating money (Gyldensted 2011; McIntyre 2015). Moreover, the study provides support for Pals’ (2006) notion that narrative completion of a story (in this study via solution-based information) helps to diminish negative emotional responses. Also, in line with the broaden-and-build theory (Fredrickson 1998, 2001), the positive emotions that were included in the constructive newscast may have played a stimulating role in improving emotional and prosocial responses. We need to note that the effect sizes that were found are rather small, which is quite common in this kind of studies on media effects (c.f., Wilson and Sherrell 1993). Although this implies that better insight into how constructive elements, among other factors that are not incorporated here, can be beneficial for preadolescents’ responses to news is warranted, introducing positive psychology to news can be seen as a promising development.

With regard to the conclusions, a few nuances should be made. First, in contrast to our expectation, preadolescents’ prosocial intentions did not differ between constructive and nonconstructive news directly after exposure. This might be explained by the fact that preadolescents in the constructive condition were exposed only once to two items containing prosocial content (item 1 and item 3) embedded within the entire newscast. Prior research suggests that a change in prosociality is more likely when either being exposed to such content repeatedly or for a longer period of time (Mares 2013; Mares and Woodard 2012). The finding that discussing the constructive newscast, which included prosocial content, enhanced prosocial intentions provided preliminary support for this. By talking about it, the prosocial content was reinforced via peers, which may explain the further increase in prosocial intentions.

Second, contrary to the expectation, we found that peer discussions did not improve emotional responses of preadolescents who watched constructive news. It appeared to be beneficial for preadolescents in the nonconstructive condition only to discuss with peers what they saw and experienced. The lack of change in emotions in the constructive condition might be due to the fact that preadolescents in the constructive condition already reported higher levels of positive emotions directly after watching the newscast, compared to preadolescents in the nonconstructive condition. Since it is an important characteristic of constructive journalism to provide the most important information about the event (c.f., Gyldensted 2015), the constructive broadcast contained basic information about the disaster reported in a rather negative manner. It is, therefore, unlikely to expect that positive emotional responses to this constructive story can become rather high. It might be that preadolescents reached their optimal level of positive emotions already after watching the constructive broadcast, which makes a further increase in positive emotions after peer discussion unlikely.

With regard to negative emotions, the content of the peer discussion may have hindered a further decrease in negative emotional feelings. We observed during the experiment that preadolescents in all conditions were impressed by the havoc that was shown in the opening of the newscast. Consequently, this was a prominent part of the peer discussions in both the constructive and the nonconstructive condition. To be more specific, each group was asked to write down the three most important ideas regarding the newscast they just saw and discussed. A preliminary analysis of these answers showed that in the constructive condition, 86.55% of the answers had a negative connotation (e.g., words as shocking, sad for Japan, and tsunami were frequently mentioned), whereas only a few constructive words (e.g., helping, future, fund raising) were mentioned. For preadolescents in the constructive condition, this implies that the positive elements that were present in the news were less prominently (or sometimes not) discussed. Thus, the peer discussion particularly may have reinforced the negative emotions that preadolescents already experienced after watching the constructive stories, which may explain why discussing the constructive newscast did not further improve their emotional responses to news. We need to note here that also preadolescents in the nonconstructive condition discussed the negative elements of the newscast about the tsunami in Japan (90% of their answers were negative), but as their negative emotions increased quite substantial due to the exposure to the news (post-exposure measure), a beneficial effect after peer discussion (follow up measure) is easier to reach. This may explain why a positive effect of peer discussion was found in the nonconstructive condition. To get more insight into this, taking a closer look at the entire content of peer discussions may shed more light on the question why and how peer discussions can moderate the effects of news on preadolescents. In line with that, it may be interesting to investigate the influence of qualitative good vs. bad discussions.

There are several limitations that should be mentioned. First, preadolescents were only exposed to one kind of topic, a natural disaster, within the broad spectrum of negative news topics that frighten preadolescents (c.f., Riddle et al. 2012; Walma van der Molen et al. 2002). It would be interesting to take a closer look at other topics as well, especially to make the findings of the current study more generalizable. It may particularly be interesting to investigate effects of news stories that happened in the closer surrounding of the participants. Vividness theory predicts that vivid information—i.e., information that is “emotionally interesting, concrete, and imaginary-provoking, and proximate in a sensory, temporal, or spatial way” (Nisbett and Ross 1980, p. 45)—has more influence on viewer responses than less vivid information (Zillmann and Brosius 2000). As the 2011 tsunami in Japan was less vivid for the Dutch preadolescents in our study, future research could investigate whether more vivid news would have a stronger effect on preadolescents’ reactions. Moreover, it would be relevant to investigate responses to other topics than natural disasters, because there might be a difference in the possibilities to frame negative events in a more constructive way and in the extent to which such stories can affect particularly prosocial intentions. For example, crime news can inform the audience about recent incidents in which people got injured or killed by an act of an individual. It might be harder to present solutions in such stories, and it may also be difficult to act in a prosocial manner as a response to this news. This supports the necessity to investigate whether and how constructive elements can be included in other negative topics beyond natural disasters and to investigate how this may inspire prosociality.

Second, studies that test how elements from positive psychology in news stories affect (young) audiences are scarce. In particular, this has led to limitations in conceptualizing constructive news reporting. Because the domain of constructive journalism is quite new, the conceptualization of constructive news reporting is still in its infancy. More research is needed to examine potential underlying processes to explain why and how constructive news reporting influences peoples’ emotional reaction to news. Moreover, it is worth exploring whether there are other elements from positive psychology than solution-based reporting and positive emotions only that are able to reduce negative responses to news and to promote prosocial intentions. Gaining more insight and developing a more detailed conceptualization of constructive journalism may support the promising effects of introducing positive psychology to news.

Third, the influence of peer discussion has been studied in a rather exploratory manner in the current study. Due to the design of the study, we cannot rule out that the effects are at least partly explained by time. To be more specific, the preadolescents in the peer discussion condition answered the final questions (follow up measure) about 10 min later than preadolescents who did not participate in a discussion (post-exposure measure). It might be that the longer time frame between exposure to the news and the final questionnaire affected their emotions and intentions. However, because of the differences that were found between constructive and nonconstructive news, it is unlikely that this time difference explains all differences between the peer discussion and no peer discussion group. Thus, the first results regarding peer discussion are promising, but future research should focus on examining this phenomenon more closely.

Fourth, the sample of classes and schools was too small to control whether these factors may have influenced the results. Consequently, we do not know whether class or school factors (e.g., attention for news, classroom culture) may have played a role here. Future research should, therefore, include more classes from a larger number of schools to enable multilevel analysis procedures.

Our last remark concerns the influence of constructive journalism on memory. Although the basic information in the constructive vs. the nonconstructive newscast was comparable, we did not investigate whether preadolescents actually remembered the most important information to the same extent. As it is journalists’ primary goal to inform citizens about events in society (c.f., McIntyre 2015), it would be of great value to investigate memory after having watched either constructive or nonconstructive news, in order to test whether a constructive style of reporting still adhere to the informative function of news.

## Conclusion

The findings of the current study revealed that both constructive news reporting and peer discussion could function as tools to make news less harmful for preadolescents. Furthermore, because constructive news is able to enhance positive emotions and to decrease negative emotions in preadolescents, the number of preadolescents suffering from anxiety-related problems, such as nightmares after having watched negative news, could be reduced. We therefore argue that news programs aiming at preadolescents, which are broadcast in several countries across the world (c.f., Alon-Tirosh and Lemish 2014), should consider using elements from positive psychology more often. Moreover, researchers should expand the emerging field of research on constructive news. The study results might also contribute to educational practice. As some beneficial effects of peer discussion were found, schoolteachers could be advised to facilitate peer discussion of frightening topics in the news after their pupils have watched it. The results of the current study could also stimulate parents to let their children watch the news together with peers (e.g., friends or siblings of comparable ages) and to motivate that they talk about it. In all, by combining constructive elements in newscasts and a following peer discussion, preadolescents’ negative emotions may be reduced and prosocial intentions may be increased. This may encourage the well-being of preadolescents, people in their close environment, and ultimately society.

## Footnotes

1 For exploratory purposes, we also measured enjoyment (post-exposure and follow up) by asking preadolescents to indicate on a 4-point scale (ranging from 1 = *totally disagree* to 4 = *totally disagree*) how exciting, delightful, boring, stupid, enjoyable, and dull they experienced the news program. Principal component analyses indicated that only the items “delightful” and “enjoyable” together formed a discernible dimension. Analyses with these two items showed that there were no differences in enjoyment after watching either constructive or nonconstructive news. Moreover, peer discussion did not affect enjoyment. Since this investigation was exploratory, and because of limitations in the measurement of enjoyment, we decided to exclude “enjoyment” as dependent variable in the study.
